# ACE2 activation protects against cognitive decline and reduces amyloid pathology in the Tg2576 mouse model of Alzheimer’s disease

**DOI:** 10.1007/s00401-019-02098-6

**Published:** 2020-01-25

**Authors:** Charles E. Evans, James S. Miners, Giulia Piva, Christine L. Willis, David M. Heard, Emma J. Kidd, Mark A. Good, Patrick G. Kehoe

**Affiliations:** 1 School of Psychology, Cardiff University, Cardiff, CF10 3AT UK; 2 School of Pharmacy and Pharmaceutical Sciences, Cardiff University, Cardiff, CF10 3NB UK; 3 Dementia Research Group, Translational Health Sciences, Bristol Medical School, University of Bristol, Level 1 Learning and Research, Southmead Hospital, Bristol, BS10 5NB UK; 4 School of Chemistry, University of Bristol, Cantock’s Close, Bristol, BS8 1TS UK

**Keywords:** Alzheimer’s disease, Renin–angiotensin system, ACE2, MasR, DIZE, Angiotensin-(1–7)

## Abstract

Mid-life hypertension and cerebrovascular dysfunction are associated with increased risk of later life dementia, including Alzheimer’s disease (AD). The classical renin–angiotensin system (cRAS), a physiological regulator of blood pressure, functions independently within the brain and is overactive in AD. cRAS-targeting anti-hypertensive drugs are associated with reduced incidence of AD, delayed onset of cognitive decline, and reduced levels of Aβ and tau in both animal models and human pathological studies. cRAS activity is moderated by a downstream regulatory RAS pathway (rRAS), which is underactive in AD and is strongly associated with pathological hallmarks in human AD, and cognitive decline in animal models of CNS disease. We now show that enhancement of brain ACE2 activity, a major effector of rRAS, by intraperitoneal administration of diminazene aceturate (DIZE), an established activator of ACE2, lowered hippocampal Aβ and restored cognition in mid-aged (13–14-month-old) symptomatic Tg2576 mice. We confirmed that the protective effects of DIZE were directly mediated through ACE2 and were associated with reduced hippocampal soluble Aβ_42_ and IL1-β levels. DIZE restored hippocampal MasR levels in conjunction with increased NMDA NR2B and downstream ERK signalling expression in hippocampal synaptosomes from Tg2576 mice. Chronic (10 weeks) administration of DIZE to pre-symptomatic 9–10-month-old Tg2576 mice, and acute (10 days) treatment in cognitively impaired 12–13-month-old mice, prevented the development of cognitive impairment. Together these data demonstrate that ACE2 enhancement protects against and reverses amyloid-related hippocampal pathology and cognitive impairment in a preclinical model of AD.

**Electronic supplementary material:**

The online version of this article (10.1007/s00401-019-02098-6) contains supplementary material, which is available to authorized users.

## Introduction

Overactivity of the classical renin–angiotensin system (cRAS) within the brain has been implicated in the pathogenesis of Alzheimer’s disease (AD) (reviewed by [1, 2]). Elevated ACE1 activity and higher levels of Ang-II and the angiotensin-II type 1 receptor (AT1R) have been reported in human AD brain tissue [3–6]. Evidence from large epidemiological and clinical studies indicate that some cRAS-targeting anti-hypertensives, including ACE1 inhibitors (ACE-Is) and AT1R receptor blockers (ARBs), reduce the incidence of AD [7–9], and can prolong the conversion to MCI and delay the onset of dementia [10–12]. Clinical use of anti-hypertensives is associated with improved tau and Aβ indexes in CSF and autopsied brain tissue [11, 13, 14]. Insights from animal models indicate that the protective effects of ACE-Is and ARBS are associated with anti-inflammatory and anti-oxidative stress mechanisms, in addition to lowering blood pressure and improving cerebral blood flow [15–17]. Clinical trials are currently underway to determine if cRAS-targeting anti-hypertensives will have a beneficial effect in patients with AD [18, 19].

Activity of the cRAS is counterbalanced by downstream regulatory RAS (rRAS) pathways [20, 21], which appear to be the dominant RAS pathways in the brain [22, 23]. Enzymes such as ACE2 and aminopeptidases-A and -N convert Ang-II (and Ang-III) into smaller bioactive fragments, most commonly Ang-1–7 and Ang-IV, reducing Ang-II associated cRAS signalling via AT1R. Recent findings show reduced CNS activity in the rRAS pathway and that induction of ACE2, or Ang-(1–7) activation of MasR signalling, is neuroprotective against ischaemic stroke and neurogenic hypertension in experimental animal models of CNS injury [24, 25]. ACE2 overexpression is associated with reduced cRAS signalling, including reduced ACE1 activity, Ang-II level and AT1R expression [26–29] and reduced oxidative stress and neuro inflammation. In a recent comparative study, rRAS activation outperformed a prototype cRAS blocker in reducing oxidative and inflammatory responses in an ischemia/reperfusion mouse model of stroke [30]. Activation of downstream rRAS receptors, such as MasR, insulin-regulated aminopeptidase (IRAP) and c-MET, which are primarily expressed in the hippocampus [31], all induce long-term potentiation (LTP) and knockout of MasR disrupts object recognition memory in rodents [32, 33] and reviewed by [34]. Collectively these data suggest that rRAS activation offers protection against many of the pathological disease processes associated with cRAS activation that are characteristic in AD but may in addition directly benefit normal cognition, through promoting synaptic plasticity processes linked to memory.

We have previously shown that there is dysregulation of endogenous rRAS activity in postmortem confirmed AD brain tissue, which is strongly associated with cRAS overactivation and disease pathology (Aβ and Tau) [35, 36]. Specifically, ACE2 activity, predominantly involved in the conversion of Ang-II to Ang-(1–7) [37, 38], was reduced by almost 50% in AD cases, which was significantly related to Aβ and Tau levels [35]. In an ovariectomised D-galactose rodent model of ageing and dementia, enhancement of ACE2 activity by administration of DIZE reduced brain Aβ pathology and improved cognitive performance [39]. We now present the first data of the impact of ACE2 enhancement in an established transgenic APP mouse model, Tg2576 mice, in which the timing and onset of Aβ pathology and behavioural abnormalities have been well characterised and recognised to model abnormalities in human AD. This study tested the hypotheses that enhancement of ACE2, using an established ACE2 activator (DIZE), will reduce Aβ-related pathology and restore cognitive function in symptomatic aged Tg2576 mice and prevent the onset of cognitive decline when administered to pre-symptomatic (younger) Tg2576 mice.

## Materials and methods

### Animals

A total of 182 male mice were used in this study across four separate cohorts (see Table 1). All WTs and Tg2576 mice within each individual cohort were from the same litters to act as appropriate littermate controls. Mice were individually housed due to their aggressive behaviour, as previously reported [40, 41]. Tg2576 mice expressing the human *APP*^*695Swe*^ mutation were maintained on a hybrid C57Bl/6 × SJL background [42]. Mice were housed on a 12-h light–dark cycle. Testing occurred during the light period (08:00–19:00). Mice were allowed *ad libitum* access to food and water throughout the duration of the experiment. All experiments were conducted in compliance with the UK Home Office under the Animal Scientific Procedures Act (1986) and EU regulations.

**Table 1:** Numbers of mice used in each experiment. The age at which mice commenced and finished behavioural assessment and were culled is shown

**Cohort 1 (30 Day DIZE administration)**						
WT Vehicle	Tg2576 Vehicle	Tg2576 DIZE	-	Total	Age at Start	Age at End
12	11	9	-	32	13.5 months	15 months

**Cohort 2 (30 Day DIZE and DIZE+C16 administration)**						
WT Vehicle	Tg2576 Vehicle	Tg2576 DIZE	Tg2576 DIZE+C16	Total	Age at Start	Age at End
21	12	13	11	57	13.5 months	15 months

**Cohort 3 (30 Day DIZE administration; Synaptosome Analysis**						
WT Vehicle	Tg2576 Vehicle	WT DIZE	Tg2576 DIZ	Total	Age at Start	Age at End
7	7	7	7	28	13 months	15 months

**Cohort 4 (10-Week and 10-Day administration)**						
WT Vehicle	Tg2576 Vehicle	Tg2576 DIZE (10-week)	Tg2576 DIZE (10-day)	Total	Age at Start	Age at End
20	16	11	17	64	9 months	12 months

### Drugs

Diminazene aceturate (DIZE) was purchased from Santa-Cruz Biotechnology (CAS 908-54-3). DIZE was freshly prepared daily in 0.9% sterile saline to a concentration of 50 mg/ml. DIZE was administered to Tg2576 mice at 15 mg/kg/day by intraperitoneal (i.p.) injection using a BD MicroFine™ 0.5-ml insulin syringe. All other mice received equivalent volumes of vehicle (saline) injections. This dose was based on several previous studies showing that the optimal dose of DIZE in inducing ACE2 activity was 15 mg/kg/day [39, 43, 44] and a preliminary study of our own across a range of DIZE concentrations by IP that confirmed previous findings (data not shown).

A competitive ACE2 antagonist, based on the structure of a commercially available compound, C16 (MLN4760, Merck Millipore) was synthesised according to reported procedures [45], and purified by reverse phase flash chromatography. Purity and identity were confirmed by ^1^H and ^13^C NMR spectroscopy, polarimetry, high-resolution mass spectrometry and HPLC. The compound data were in good accordance with the literature and the inhibitor performed similar to the commercial inhibitor when tested in vitro using recombinant mouse ACE2 (R&D systems) (data not shown). The ACE2 inhibitor was co-administered with DIZE in relevant experiments, in an identical vehicle as DIZE, at 25 mg/kg/day, which was the dose most consistently used in previous C16 studies [46, 47].

### Experimental design

**Fig. 1:**
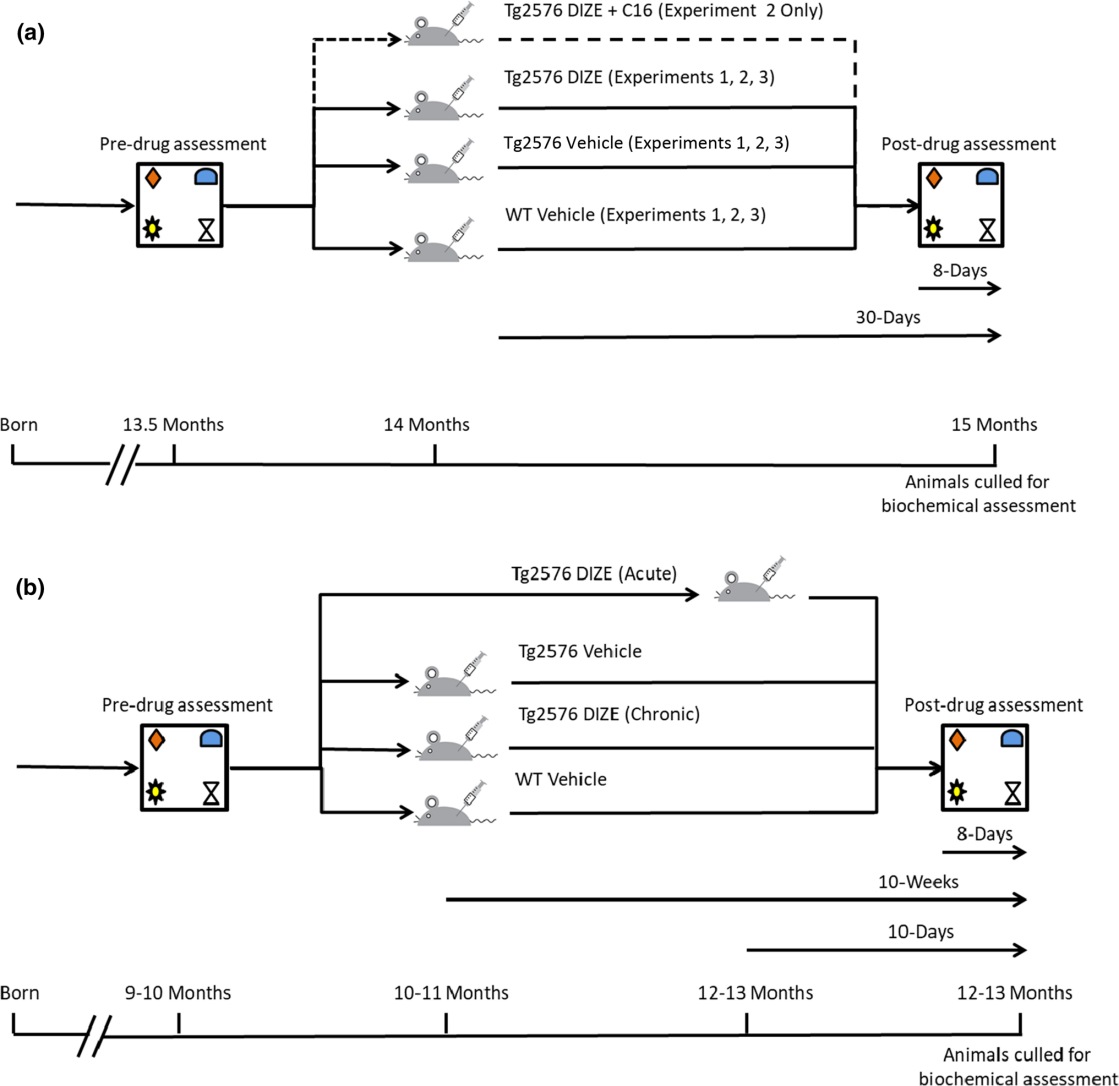
Schematic representation of the experimental design in **a** for Experiments 1, 2 and 3 and **b** for Experiment 4. **a** Male mice between 13 and 14 months of age were subject to pre-drug assessment in the object-in-place (OiP)-associative recognition memory task to establish a baseline measurement. Mice were then randomly assigned into groups to ensure that there was no significant difference in behaviour prior to treatment. All WT mice were administered vehicle (saline) only. In Experiment 1, Tg2576 mice were administered either vehicle (saline) or DIZE (15 mg/kg/day) for 30 days. In Experiment 2, a third Tg2576 group was added that was administered DIZE + C16 (25 mg/kg/day) for 30 days. In Experiment 3, both WT and Tg2576 groups were administered vehicle or DIZE. In each experiment, on day 23 of treatment, post-drug assessment commenced with habituation and testing was performed on days 28 and 30. All mice were culled and brains were dissected on day 30 immediately following post-testing and underwent pathological and biochemical assessment. **b** Mice underwent pre-drug assessment at 9–10 months of age and all mice were found to be asymptomatic. The mice were then divided into four groups: WT vehicle, Tg2576 vehicle, Tg2576 DIZE (chronic) and Tg2576 DIZE (acute). Tg2576 groups were allocated based on OiP performance so that all groups showed comparable levels of contact time and DR score performance. All groups except Tg2576 DIZE acute commenced IP DIZE treatment (15 mg/kg/day) or vehicle for 10 weeks. At 8 days prior to the final two test days at 12–13 months of age, DIZE acute mice received drug treatment daily for 10 days (15 mg/kg/day). Tg2576 mice had previously been shown to be cognitively impaired at 12–13 months of age in Experiments 1 and 2. Mice across all four groups were re-tested at 12–13 months of age. At the end of the study, after all mice had undergone OiP testing, they were culled immediately and underwent pathological and biochemical assessments

In Experiment 1, we investigated whether DIZE influenced cognitive performance and Aβ-related pathology in male 13–15-month-old Tg2576 mice. An outline of the experimental design is shown in Fig. 1a. Mice from cohort 1 consisted of three groups: WT vehicle (*n* = 13), Tg2576 vehicle (*n* = 11) and Tg2576 DIZE (*n* = 11). Prior to group allocation, all mice were tested on the object-in-place (OiP) task (as described below) to establish a baseline of cognitive performance and to ensure that the Tg2576 groups had a comparable level of performance prior to drug administration. Following group allocation, all mice were weighed and six mice/group were used to assess mean arterial blood pressure (MABP) and heart rate (HR) at baseline (see also below). A 30-day DIZE/vehicle treatment period then commenced. Mice received daily i.p. injections in the morning and were rested for at least 1 h before any physiological/behavioural testing was performed. Mice were weighed and MABP and HR measurements were recorded weekly in the sub-groups of mice until the end of the treatment period. Behavioural assessment was again conducted starting with habituation on day 23 of drug treatment followed by the OiP task on day 28 and day 30 of treatment. Immediately following behavioural assessment at day 30, the mice were culled by cervical dislocation and the left and right hippocampi and cortices were dissected and snap frozen in liquid nitrogen and stored at − 80 °C until processed for downstream biochemical and pathological assessment. A total of three mice in cohort 1 died during the Experiment 1**—**one WT vehicle and two from the Tg2576 DIZE group (one on day 2 of administration and the second on day 29). Therefore, the final n for the WT vehicle group was *n* = 12, the Tg2576 vehicle group remained at *n* = 11 and the Tg2576 DIZE group was *n* = 9.

In Experiment 2, we aimed to (1) replicate our findings from Experiment 1 in a larger independent cohort (cohort 2) and (2) determine if the observed protective effects of DIZE were specific to ACE2 enhancement. The experimental design used identical dosing and behavioural testing methods as outlined for Experiment 1 (see Fig. 1a). However, in this experiment, we included an additional group of mice that were co-administered DIZE and C16 (an ACE2 inhibitor) to test the hypothesis that enhanced ACE2 activity was required for the protective effects of DIZE. A total of four mice died in cohort 2 (two mice administered DIZE and two administered DIZE + C16). Final group numbers in cohort 2 were, therefore, as follows: WT vehicle (*n* = 22), Tg2576 vehicle (*n* = 11), Tg2576 DIZE (*n* = 12), Tg2576 DIZE + C16 (*n* = 11). Cohorts 1 (Experiment 1) and 2 (Experiment 2) were used for discovery and replication studies, respectively, to test and verify the behavioural changes independently but brain tissue was collected and combined from both cohorts, given the identical experimental dosages and design, to allow for meaningful statistically powered biochemical assessment of changes in ACE2, Aβ, cytokines, and markers of astrocyte, microglial and vascular function.

In Experiment 3, we studied a separate cohort of mice (cohort 3) consisting of four groups: WT vehicle (*n* = 7), WT DIZE (*n* = 7), Tg2576 vehicle (*n* = 7) and Tg2576 DIZE (*n* = 7) (all 13–15 months old). The addition of WT DIZE group was designed to assess if DIZE affected WT mice behaviour and biochemistry in the same way as Tg2576 mice. The experimental procedure replicated that of Experiments 1 and 2. Upon completion of this study, mice brains were either snap frozen for biochemical assessment or processed for IHC and IF analyses.

In Experiment 4, we used a 4th group of mice (cohort 4) to investigate if long-term (10 weeks) DIZE administration (Tg-DIZE chronic) could protect against the development of Aβ pathology and cognitive impairment when administered to pre-symptomatic younger mice aged 9–10 months. To validate and extend our observations on the beneficial effects of DIZE in aged (symptomatic) Tg2576 mice, we compared the chronic treated (10 weeks) condition with a group that received DIZE acutely for 10 days (Tg-DIZE acute) at 12–13 months of age (*n* = 17) immediately prior to behavioural testing and tissue collection. The experimental procedure is summarised in Fig. 1b. We studied WT vehicle (*n* = 20), Tg2576 vehicle (*n* = 16) and Tg-DIZE chronic (*n* = 11) or Tg-DIZE (*n* = 17).

### Behavioural assessment

All mice were tested on the OiP task as previously described [48, 49]. A detailed description of the OiP task is presented in Supplementary Fig. 1, online resource. Briefly, prior to testing associative recognition memory, animals were habituated to the arena. Mice were allowed to explore the arena freely for 10 min on day 1 and day 2. Mice were then further habituated for two consecutive days to the arena containing four different objects for 10 min each day. Each object was approximately 15 cm from the walls of the arena and 25 cm apart from each other. This arrangement remained constant throughout the study. A different set of objects was used each day and across both time points tested. No sets of objects were re-used during habituation or testing. Each mouse received two rounds of behavioural testing; each test was separated by 48 h. The group of objects used and specific pair of objects that underwent a location switch and their order across days were counterbalanced within and between groups.

Time spent exploring the objects was recorded in both sample and test phases. Object exploration was defined according to the methods described previously by Ennaceur and Delacour (1988). In brief, object contact was defined as when an animal was within a 2 cm radius of the object and directly facing, sniffing, gnawing, but not climbing or sitting on, the objects. A discrimination ratio (DR) was used to provide an index of the mouse’s discrimination performance in the test phase that was independent of individual differences in object contact times; this was calculated as the time spent exploring objects in novel locations/the time spent exploring all objects. All reported contact times and DR scores were averaged across the two trials. We mitigated against the potential effects of repeat testing in our study by counterbalancing across object sets and switching the location of objects within and between groups. All objects were cleaned before each phase of testing to reduce the use of odour cues introduced from handling the objects.

### Blood pressure measurement

Our aim was to use a dose of DIZE that influenced ACE2 activity but did not change blood pressure. The effect of DIZE (15 mg/kg/day) on MABP and HR was, therefore, assessed at weekly intervals over the entire course of the study in Experiment 1. Measurements were taken prior to DIZE treatment to determine a baseline MABP and then at weekly intervals until the end of the study. Six mice from each treatment group were habituated to the apparatus (Harvard Apparatus) and used throughout the study. To habituate animals for MABP and HR recordings, each mouse was given a 15-min trial/day for 4 days. Mice were placed in a mouse holder (Part no. 76-0184) underneath a heating unit (Part no.76-0178) in a dark room. The tail cuff and pulse transducer (Part no. 76-0432) were placed around the mouse’s tail and inflated 7–8 times/session. Following habituation, each mouse was placed in the holding unit and given 2–3 min to adapt to the dark room and warmth from the heating device, after which the tail cuff was inflated and MABP and HR was recorded from the blood pressure recording unit (Part no. 76-0173). Three recordings (that were averaged) were taken per mouse. Mice were then removed from the holding unit and returned to home-cages, after which the holding units were wiped clean with 70% ethanol wipes and allowed to dry thoroughly between mice, to prevent stress that may have been caused from residual scents from other males.

### Biochemical measurements of markers of disease pathology

Immediately following behavioural testing, mice were culled by cervical dislocation and their brains were removed and dissected. In Experiments 1, 2 and 4, both hemispheres were dissected into cortex, hippocampus and frontal cortex and snap frozen at − 80 °C until protein extraction as previously described [51]. In Experiment 3, the left hemisphere was dissected as above, snap frozen, and used for synaptosome extraction (see below). The contralateral hemisphere was fixed in 4%PFA in 0.1 M PBS for 12 h before being transferred to 25% sucrose in PBS for 48 h. Brains were then sliced using a vibratome and stored in cryoprotectant as previously described [52].

#### Synaptosome extraction

Synaptosome extractions were performed using Syn-PER™ synaptic protein extraction reagent (ThermoFisher, UK). Western blotting was performed using standard methods as described previously [51]. Briefly, after protein quantification, 20 μg protein/sample was resolved on either a 10 or 7.5% polyacrylamide gel and detected with the relevant antibody (NR2B, pY1472-NR2B (Millipore), GluA1, pSer845 GluA1, PSD95 (Abcam), total ERK, phospho-ERK (Cell Signalling Technologies), NR1 (BD Biosciences), Total Tau (DAKO), PHF1 (p-Tau Ser396/404; a generous gift from P. Davies) and β-actin (Sigma).

### Amyloid-β ELISA measurements

Soluble and insoluble fractions were extracted from right hippocampus and right cortex as previously described [53]. Briefly, brain samples of each mouse were homogenised in 2% sodium dodecyl sulphate (SDS) using a Precellys 24‐Dual (Bertin technologies). Homogenate was centrifuged at 28,300 rpm for 1 h at 4 °C. The supernatant was carefully removed and stored at – 20 °C for later analysis as the “soluble” fraction. The insoluble pellet was further dissolved in 70% formic acid. Samples were centrifuged again at 28,300 rpm for 1 h at 4 °C. The supernatant was carefully removed and added 1:20 to a neutralising buffer (1 M Tris, 0.5 M Na_2_HPO_4_, pH 11) and stored at − 20 °C for subsequent analysis.

Enzyme-linked immunosorbent assay (ELISA) kits specific for human Aβ40 and Aβ42 (Invitrogen:#KHB3482 and #KHB3441) and Aβ43 (Tecan: RE59711) were used to quantify soluble and insoluble Aβ species according to the manufacturer’s instructions. Results were expressed as picograms (pg) per milligram (mg) of tissue. The mean from duplicate measurements are presented.

### ACE1 and ACE2 enzyme activity measurements

To quantify the enzyme activity of ACE1 and ACE2, we used established fluorogenic assays that have previously been described [4, 5, 35, 54]. Brain tissue samples from the left hippocampus and left cortex were dissected and homogenised in 1% SDS lysis buffer using an automated Precellys tissue homogeniser. ACE1 and ACE2 activities were quantified using an ACE1 fluorogenic peptide Abz-FRK(Dnp)-P (Enzo Life Sciences) in the presence/absence of captopril (Enzo) or an ACE2 substrate peptide Mca-APK(Dnp) in the presence/absence of MLN4760 (Millipore). Specific enzyme activity was calculated by subtracting the fluorescence in the presence of the specific inhibitor from total fluorescence without inhibition.

#### ACE2 protein level measurement

Total protein levels of ACE2 were measured in the right hippocampus by ELISA according to the manufacturer’s instructions (Abcam: ab213843).

#### Angiotensin-I, -II and -(1–7) measurement

Levels of Ang-I, Ang-II, Ang-(1–7) were measured in hippocampal homogenates prepared in 1% SDS using in-house direct ELISA’s, as previously described for the quantification of peptides in human postmortem brain tissue and CSF [4, 5, 35, 36, 54]. In brief, serial dilutions of recombinant Ang-I, Ang-II or Ang-(1–7) (5000–78.125 pg/ml) (Abcam, Cambridge, UK), and mouse brain extracts (diluted 1 in 20 in PBS) were coated on a Nunc Maxisorp 96-well plate (ThermoFisher Scientific, Waltham, MA, USA) for 2 h at room temperature on a plate shaker (300 rpm), washed, and incubated with biotinylated detection antibodies [biotinylated anti-Ang-I (diluted 1 in 100 in PBS); biotinylated anti-Ang-II (diluted 1 in 500 in PBS) or biotinylated anti-Ang-(1–7) (diluted 1 in 100 in PBS) (all from Cloud-Clone, Wuhan, China)] for another 2 h with shaking. After washing and incubation with streptavidin:HRP (diluted 1 in 200 in 0.1% PBS:Tween-20) (R&D systems) for 20 min, the plates were washed and incubated with TMB substrate (R&D systems). The reaction was stopped after 20 min and absorbance was read at 450 nM using a FLUOstar OPTIMA plate reader (BMG Labtech, Aylesbury, BUCKS, UK). Samples were measured in duplicate and interpolated from the serial dilution of recombinant protein.

#### Cytokine measurements

ELISA kits specific for mouse interleukin (IL)-1β, IL-6 and IL-10 and TNF-α (R&D Systems: DY401, DY406, DY417, DY410) were used to quantify cytokine levels in hippocampal soluble sample extracts. Results were expressed as pg/mg. Means from duplicate readings are presented.

#### Microglial (CD68 and IBA1) and astrocyte (GFAP) ELISA

The level of an astrocytic activation marker (GFAP) and two independent microglial markers (IBA1 and CD68) were measured in hippocampal samples homogenised in 1% SDS buffer using commercial ELISA kits following the manufacturer’s protocols: GFAP (samples diluted 1 in 2000) (Cat no: SEA068Mu), IBA1 (samples diluted 1:200 in PBS) (Cat no. SEC288Mu) (Cloud Clone, Wuhan, China) and CD68 (samples diluted 1 in 650 in PBS) (Cat no. OKEH03503, Aviva Systems Biology, San Diego, USA).

### Vascular markers of tissue oxygenation and BBB leakiness

The level of VEGF was measured in hippocampal lysates diluted 1 in 40 in PBS using a commercially available ELISA kit (Cat no. DY493, R&D systems) (MA, USA). The ratio of MAG:PLP is a marker of tissue oxygenation previously developed in studies using human postmortem brain tissue [55, 56]. MAG was measured in hippocampal lysates diluted 1 in 650 in PBS using an in-house direct ELISA that showed species cross-reactivity in mice as previously described [55, 57]. PLP concentration was measured in hippocampal samples diluted 4 in 100 in PBS using a commercially available sandwich ELISA following the manufacturer’s instructions (Aviva Biological Systems, San Diego, USA).

### Immunofluorescent labelling

Brains hemispheres were fixed in 4% paraformaldehyde in 0.1 M PBS/PFA for 12 h at room temperature. Brains were then transferred to 25% reagent-grade sucrose in 0.1 M PBS. Each brain remained in sucrose until sinking, indicating it was fully saturated (approximately 48 h), and was then sliced using a vibratome. 40-μm coronal sections of hippocampus were stored at − 20 C in an ethylene–glycol-based cryoprotectant (Sigma, UK) until they were used for immunohistochemical analysis.

#### Parenchymal Aβ imaging

Sections were washed in 3 × 5-min changes of PBS and 3 × 5-min washes in distilled water before being incubated for 10 min in formic acid. Sections were then washed 3 × 5 min in distilled water and equilibrated in PBS before being blocked for 20 min in 5% PBS/donkey serum (Sigma-Aldrich, Dorset, UK). Sections were incubated at 4°C overnight with mouse anti-human Aβ (4G8) at 1 in 250 in PBS (Biolegend, San Diego, CA, USA). Sections were washed 3 × 5 min in PBS and incubated at room temperature for 1 h with Alexa Fluor 488 donkey anti-mouse (ThermoFisher UK) at 1 in 500 in PBS. Sections were washed 3 × 5 min in PBS and mounted using Vectashield mounting medium with DAPI (Vector Labs, Peterborough, UK).

#### MasR immunofluorescent labelling

Sections were washed in 3 × 5-min changes of PBS and blocked for 20 min in 5% PBS/donkey or goat serum (Sigma-Aldrich, Dorset, UK). Sections were incubated at 4 C overnight with rabbit anti-MasR (1:100) (Cat no. AAR-013) (Alomone Labs, Tel Aviv, Israel). Sections were washed 3 × 5 min in PBS and incubated at room temperature for 1 h (1 in 500 in PBS) with Alexa Flour 488 donkey anti-rabbit (1 in 500 in PBS) (ThermoFisher, UK). Sections were washed 3 × 5 min and mounted using Vectashield mounting medium with DAPI (Vector Labs, Peterborough, UK).

#### GFAP immunolabelling of reactive astrocytes

Sections were washed in 3 × 5-min changes of PBS and blocked for 20 min in 5% PBS/goat serum (Sigma-Aldrich, Dorset, UK). Sections were incubated at 4°C overnight with chicken anti-GFAP (1:100) (Cat no. abs4674) (Abcam, Cambridge, UK). Sections were washed 3 × 5 min in PBS and incubated at room temperature for 1 h with Alexa Fluor 594 goat anti-chicken (1 in 500 in PBS) (ThermoFisher, UK). Sections were washed 3 × 5 min and mounted using Vectashield mounting medium with DAPI (Vector Labs, Peterborough, UK).

### Statistical analysis

All statistical analyses were performed using IBM SPSS statistics (version 25). The behavioural data conformed to the assumptions of analysis of variance (ANOVA) and were analysed using a mixed measures design. Significant interactions were assessed using tests for simple main effects with Bonferroni correction. Western blot data were analysed using one-way ANOVA followed by Tukey’s post hoc analysis. ELISA data were analysed using either an independent-samples *t* test or one-way ANOVA with post hoc Tukey comparisons. All data were subject to Levene’s and Shapiro–Wilks tests for data normality prior to analysis. Appropriate transformations were carried out when necessary. Data generated from ELISA assays were quantified by comparing data to standard curves from each plate using GraphPad Prism^®^7 and normalised to total protein concentration.

## Results

### DIZE reverses associative recognition memory deficits and reduces Aβ pathology in aged Tg2576 mice

**Fig. 2:**
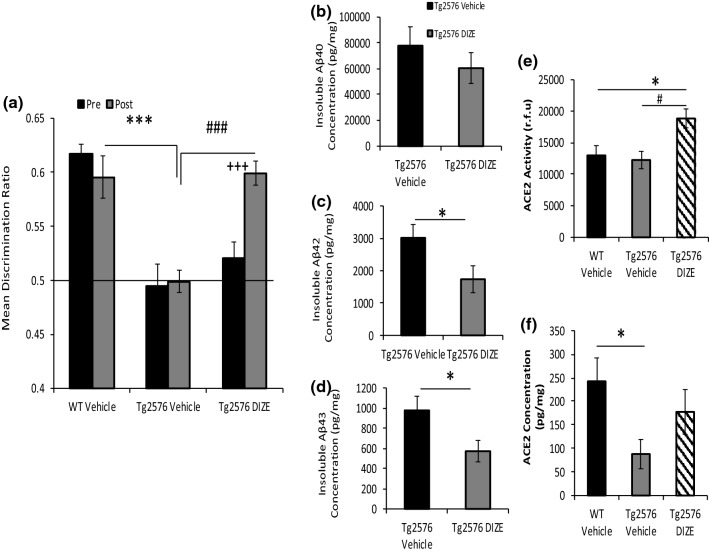
Improved associative recognition memory and reduced Aβ pathology in Tg2576 mice following peripheral administration of DIZE. **a** Following 30-day IP administration of DIZE (15 mg/kg/day), associative recognition memory, tested using the object-in-place (OiP) task, was significantly improved in Tg-DIZE mice (*n* = 9) compared to pre-DIZE scores (^+++^*p* < 0.001). Tg DIZE mice also performed significantly better than Tg vehicle mice (^###^*p* < 0.001; *n* = 11). Tg vehicle mice were impaired compared to WT vehicle mice (***p < 0.001; *n* = 12), whilst Tg-DIZE mice showed a comparable performance level to WT mice, *p* > 0.1. **b**–**d** Hippocampal insoluble Aβ40, Aβ42 and Aβ43 concentration was measured by sandwich ELISA. Insoluble Aβ42 and Aβ43 levels were significantly reduced in the hippocampus in Tg-DIZE compared to Tg-V mice (*ps < 0.05) whereas Aβ40 was unchanged. **e** Hippocampal ACE2 activity, quantified using a FRET enzyme activity assay, was significantly elevated in the hippocampus of Tg DIZE compared to WT vehicle (*p < 0.05) and Tg vehicle (p^#^ < 0.05) mice. **f** Hippocampal ACE2 level, measured by ELISA, was lower in Tg vehicle compared to WT vehicle mice (p* < 0.05); no statistically significant difference was observed between WT vehicle and Tg DIZE mice (*p* > 0.05). Behavioural data were analysed using mixed measures ANOVA. Significant interactions were further analysed by tests for simple main effects with Bonferroni corrections for multiple comparisons. Amyloid analysis was performed using independent-samples *t* test. ACE2 analysis was performed using one-way ANOVA with post hoc Tukey analysis. Error bars represent the standard error of the mean (SEM)

In Experiment 1, WT and Tg2576 mice underwent OiP testing. Contact time data were converted to discrimination ratio (DR) scores (for a full description of OiP object contact times see Supplementary Table 1, online resource). Tests for simple main effects revealed that prior to DIZE administration, both Tg2576 vehicle (Tg-V) and Tg2576 DIZE (Tg-DIZE) mice did not differ significantly but were both impaired compared to WT vehicle (WT-V) controls (*p* = 0.0001 and 0.001, respectively) (Fig. 2a). Following the 30-day treatment period, Tg-V remained impaired compared to WT-V mice (*p* = 0.0001). In contrast, Tg-DIZE mice performed comparably to WT-V mice (*p* = 1.0) and significantly better than Tg-V mice (*p* = 0.0001). The Tg-DIZE group showed no overall change in physiological parameters, including body weight, MABP and HR compared to all groups (Supplementary Fig. 2, online resource).

Hippocampal parenchymal Aβ plaque load was visualised using 4G8 immunofluorescent labelling and appeared to be lower in Tg-DIZE mice compared to Tg vehicle (Supplementary Fig. 3, online resource). To confirm our immunohistochemical observations, we measured insoluble Aβ40, Aβ42, and Aβ43 (an early-depositing and highly amyloidogenic seed that is cleaved by ACE2), in hippocampal extracts (Fig. 2c, d). Insoluble Aβ42 (*t*(18) = 2.16, *p* = 0.044) and Aβ43 (*t*(18) = 2.22, *p* = 0.04) were significantly reduced in the hippocampus of Tg-DIZE compared to Tg-V mice. Insoluble Aβ40 level was not statistically different between groups although the absolute levels were numerically lower in Tg-DIZE mice (*t*(18) = 0.89, *p* = 0.39).

Hippocampal ACE2 enzyme activity was significantly different between groups (*F*(2, 30) = 5.49, *p* = 0.01) and post hoc Tukey analysis revealed significantly elevated ACE2 activity in Tg-DIZE mice compared to WT-V and Tg-V mice (*p* = 0.028 and *p* = 0.013, respectively) (Fig. 2e). Hippocampal ACE2 protein level was also significantly different between groups (*F*(2, 30) = 3.35, *p* = 0.049) and post hoc analysis revealed a significant reduction in ACE2 protein level in Tg-V mice compared to WT-V (*p* = 0.039) but no significant difference between WT-V and Tg-DIZE (*p* = 0.56) (Fig. 2f).

### DIZE-mediated restoration of associative recognition memory in Tg2576 mice is mediated specifically through ACE2 enhancement and is associated with reduced hippocampal soluble Aβ42

**Fig. 3:**
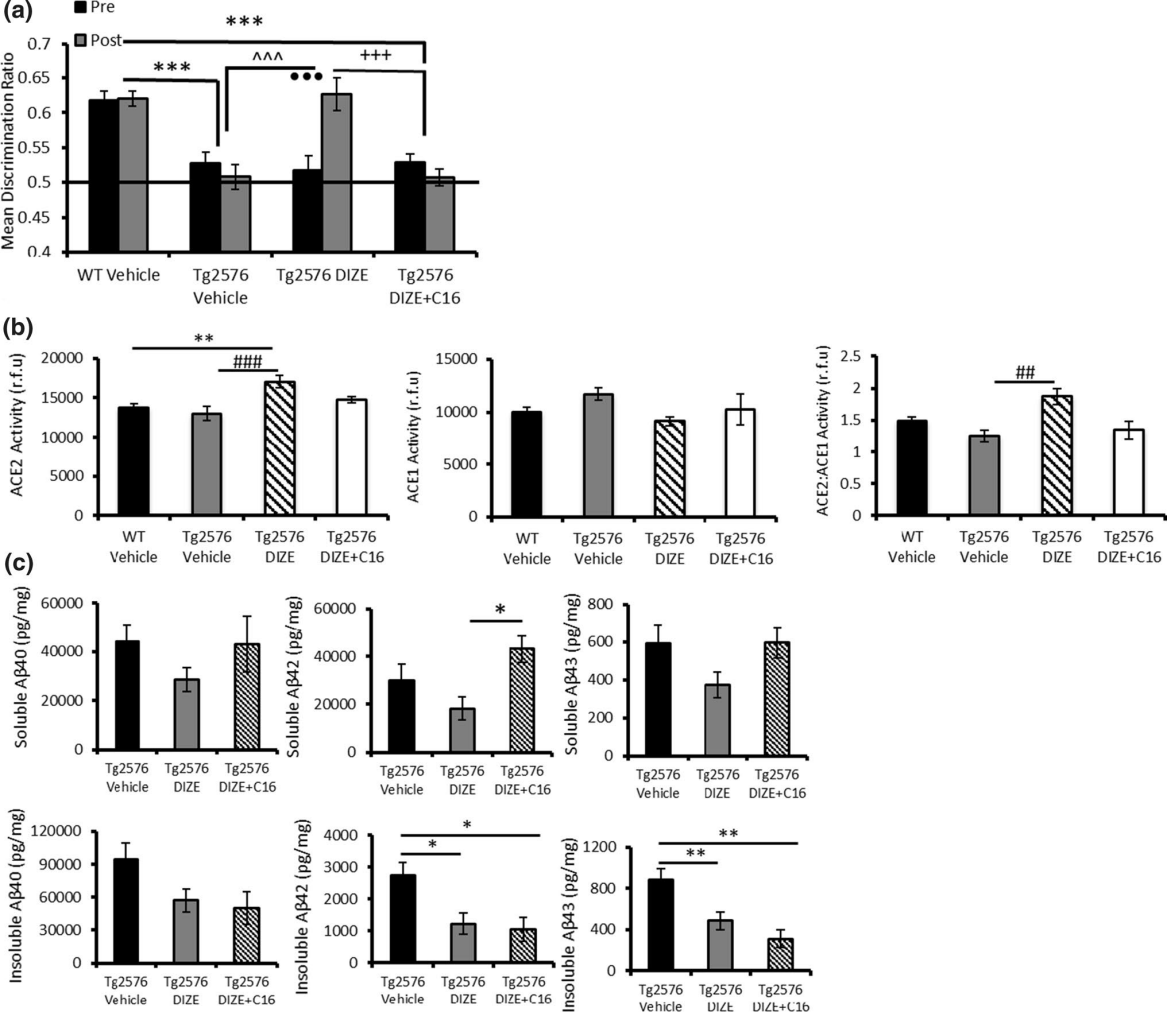
DIZE-mediated restoration of the recognition associative memory deficit in Tg2576 mice is mediated by enhanced ACE2 and is associated with reduced soluble Aβ42. **a** Associative recognition memory was impaired in Tg vehicle mice (*n* = 12) compared to WT vehicle mice (*n* = 21) (***p < 0.001) and Tg2576 DIZE-administered mice (*n* = 13) (^^^^^*p* < 0.001). DIZE improved the cognitive performance of Tg2576 mice compared to pre-DIZE administration performance (^•••^*p* < 0.001). Co-administration of DIZE + C16 in Tg2576 mice (*n* = 11) prevented the improvement compared to pre-administration performance (*p* > 0.05). DIZE + C16 mice were also impaired compared to WT vehicle (***p < 0.001) and Tg2576 DIZE mice (^+++^*p* < 0.001). **b** Hippocampal ACE2 activity was increased in Tg2576 DIZE-administered mice (*n* = 22) compared to WT vehicle (*n* = 33) (**p < 0.01) and Tg2576 vehicle mice (*n* = 23) (^###^*p* < 0.001; *n* = 23). Although numerically reduced, no difference was reported in ACE2 activity in Tg2576 mice co-administered DIZE + C16 (*p* > 0.05; *n* = 11). There was no significant difference in ACE1 activity across groups but the ratio of ACE2:ACE1 was significantly elevated in Tg2576 DIZE mice compared to Tg2576 vehicle mice (*p* < 0.01). These changes were not apparent in Tg2576 mice co-administered DIZE and C16, *p* > 0.05. **c** Soluble and insoluble levels of Aβ40, Aβ42 and Aβ43, were measured by ELISA. Soluble Aβ42 was significantly lower in Tg2576 DIZE mice compared to mice co-administered DIZE + C16 (*p < 0.05). Insoluble Aβ42 and 43 was significantly reduced in both Tg2576 DIZE mice and Tg2576 mice co-administered DIZE + C16. Insoluble Aβ40 was unchanged between Tg-DIZE and Tg-DIZE + C16 mice. Behavioural data were analysed using mixed measures ANOVA. Significant interactions were further analysed by tests for simple main effects with Bonferroni corrections for multiple comparisons. ACE2 and Aβ analysis was performed using one-way ANOVA with post hoc Tukey analysis. Error bars represent the SEM

In Experiment 2, we replicated our findings in Experiment 1 in a second cohort (cohort 2) whilst testing the hypothesis that the effects of DIZE on memory were mediated specifically through enhancing ACE2 activity. An additional group of Tg-DIZE mice was co-administered with DIZE + C16 (C16 is an ACE2 antagonist). Contact time data are presented in Supplementary Table 2, online resource. Analysis of OiP discrimination ratios (Fig. 3a) confirmed our findings in Experiment 1. Tg-V (*p* = 0.001) and Tg-DIZE (*p* = 0.001) mice were impaired compared to WT-V mice at baseline (pre-treatment). Following 30 days of treatment, Tg-V remained cognitively impaired compared to WT mice (*p* = 0.001) but memory performance in Tg-DIZE mice was significantly improved and comparable to WT-V mice. Importantly, the beneficial effect of DIZE was abolished in Tg-DIZE + C16 mice compared to Tg-V mice (*p* = 0.757) (Fig. 3a). Furthermore, Tg-DIZE mice showed better OiP performance compared to Tg-V mice (*p* = 0.001) and Tg-DIZE + C16 mice (*p* = 0.001).

As for Experiment 1, DIZE significantly altered hippocampal ACE2 activity (*F*(3, 83) = 6.42, *p* = 0.0001), which was increased in Tg-DIZE mice compared to WT-V (*p* = 0.003) and Tg-V mice (*p* = 0.001) (Fig. 3b). No change in ACE2 activity was observed in Tg-DIZE + C16 mice compared to either WT-V or Tg-V group (*p*s > 0.05). Although hippocampal ACE1 activity was numerically reduced in Tg-DIZE mice compared to Tg-V mice, it did not reach conventional levels of statistical significance (*p* > 0.05). However, the ratio of ACE2:ACE1 was significantly increased in Tg-DIZE mice compared to Tg-V mice (*p* < 0.01).

Hippocampal insoluble Aβ42 and Aβ43 levels were both significantly reduced in Tg-DIZE mice compared to Tg-V mice (*p* = 0.010 and *p* = 0.009, respectively). Hippocampal insoluble Aβ42 and Aβ43 levels also remained significantly lower in Tg-DIZE + C16 mice (*p* = 0.0018 and *p* = 0.002, respectively) compared to Tg-V mice (Fig. 3c). The same trend was observed for insoluble Aβ40 but did not reach statistical significance *F*(2, 53) = 3.026, *p* = 0.057 (Fig. 3c). All species of soluble Aβ levels were numerically lower but not significantly altered in Tg-DIZE mice compared to Tg-V (Fig. 3c) (a detailed breakdown of the percentage change in Aβ species across experimental groups is shown in Supplementary Table 5, online resource). Soluble Aβ42 was, however, significantly higher in the Tg-DIZE + C16 mice compared to the Tg-DIZE mice (*p* = 0.0037) (Fig. 3c).

### DIZE-associated cognitive improvement is associated with restored Mas receptor expression and enhanced glutamate receptor signalling in hippocampal synaptosomes

**Fig. 4:**
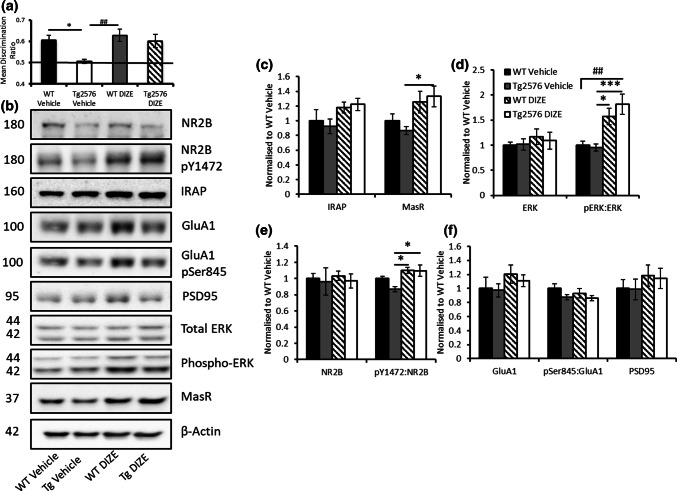
DIZE-mediated restoration of associative recognition memory in Tg2576 mice is associated with restored MasR expression and evidence of enhanced NMDA NR2B subunit glutamate signalling. **a** Tg2576 vehicle mice (*n* = 7) were impaired compared to both WT vehicle (*p < 0.05; *n* = 7) and WT DIZE mice (^##^*p* < 0.01; *n* = 7). However, Tg2576 mice administered DIZE (*n* = 7) showed comparable DR scores to both WT groups, *p*s > 0.05. **b** Representative western blot images showing altered levels of RAS receptor and markers of glutamate-mediated LTP signalling in synaptosome-enriched fractions from the hippocampus. **c** MasR level was significantly increased in Tg2576 DIZE mice compared to Tg2576 vehicle (*p < 0.05) whereas IRAP level remained unchanged. **d** To indicate changes in synaptic signalling, ERK and phospho-ERK were also probed for in hippocampal synaptosomes. No changes in total levels of ERK were reported (*p* > 0.05). However, when phosphorylated ERK was normalised to total ERK, there was a significant increase in p-ERK in Tg2576 DIZE mice compared to WT vehicle mice (^##^*p* < 0.01). There was also an increase in p-ERK in WT DIZE mice (*p < 0.05) and Tg2576 DIZE mice (***p < 0.001) compared to WT-V. **e** Total levels of NR2B were unchanged across groups (*p* > 0.05) but the ratio of phosphorylated Y1472 NR2B:total NR2B was significantly increased in WT DIZE and Tg2576 DIZE compared to Tg2576 vehicle control mice, respectively (*ps < 0.05). **f** No overall change was observed for the post-synaptic marker, PSD95, GluA1 or the ratio of pSer845:GluA1 (*p*s > 0.05). All data were analysed using one-way ANOVA with post hoc Tukey analysis. Error bars represent the SEM

In Experiment 3, we again confirmed DIZE-mediated improvement in OiP DR scores in Tg-DIZE mice in a separate cohort of mice (cohort 3) (Fig. 4a) (a detailed assessment of contact time and DR scores is shown in Supplementary Table 3, online resource). MasR was abundantly expressed in the hippocampus, specifically in the dentate gyrus and subiculum (Supplementary Fig. 4a, online resource) and appeared to be reduced in Tg-V mice compared to WT-V mice (Supplementary Fig. 4b, online resource). We were unable to quantify IF-labelling but MasR level measured by ELISA was reduced in Tg-2576 compared to WT mice (p < 0.05) (Supplementary Fig. 4c, online resource). MasR expression appeared to be increased in Tg-DIZE compared to Tg-V mice in hippocampal synaptosomes assessed by western blot (Fig. 4b). On analysis, a significant group effect was observed in the level of MasR expression, (*F*(3, 27) = 3.55, *p* = 0.029). Post hoc Tukey analysis revealed that MasR level was significantly increased in Tg-DIZE mice compared to Tg-V mice (*p* = 0.041) (Fig. 4c). In contrast, the level of the insulin-regulated aminopeptidase receptor (IRAP) (also involved in downstream rRAS signalling) was unchanged across groups (*F*(3, 27) = 1.99, *p* = 0.14).

In hippocampal synaptosomal extracts, the ratio of phosphorylated NR2B to total NR2B was altered between groups *F*(3, 27) = 5.23, *p* = 0.006 and post hoc Tukey analysis revealed that in Tg-DIZE, the ratio was increased compared to Tg-V mice (*p* = 0.012) and also in WT-DIZE compared to Tg-V mice (*p* = 0.011) (Fig. 4e). No significant change was observed in total levels of GluA1 or GluA1 phosphorylated at regulatory Ser845 (maximal effect, *F*(3, 27) = 1.35, *p* = 0.28 (pGluA1), and total levels of PSD95 were unchanged *F*(3, 27) = 0.49, *p* = 0.70 (Fig. 4f). In addition, the level of phosphorylated ERK was significantly altered, *F*(3, 27) = 8.59, *p* = 0.001. Post hoc Tukey analysis showed increased phosphorylated ERK in both Tg-DIZE (*p* = 0.001) and WT-DIZE mice (*p* = 0.032) compared to respective vehicle controls; in contrast, total ERK was unchanged across groups, *F*(3, 27) = 0.36, *p* = 0.79 (Fig. 4d) (full blots of all samples analysed are shown in Supplementary Fig. 5, online resource).

Given that tau has been shown to play a role in learning and is commonly hyperphosphorylated in AD [58, 59], we further measured levels of tau phosphorylation in hippocampal synaptosomes. However, no significant differences were observed between WT and Tg2576 mice, with or without the administration of DIZE, when comparing total levels of tau *F*(3, 20) = 0.70, *p* = 0.56 or tau phosphorylated at Ser396/404, *F*(3, 20) = 1.12, *p* = 0.37 (Supplementary Fig. 6, online resource). This may be indicative of a lack of reported tau changes in Tg2576 mice at this age and future studies should focus on tau changes in older Tg2576 mice and/or more specifically in rodent transgenic tau models.

### ACE2-mediated improvement in associative recognition memory is related to reduced inflammatory (IL-1β) but not to markers of astrocyte, microglial or vascular function

**Fig. 5:**
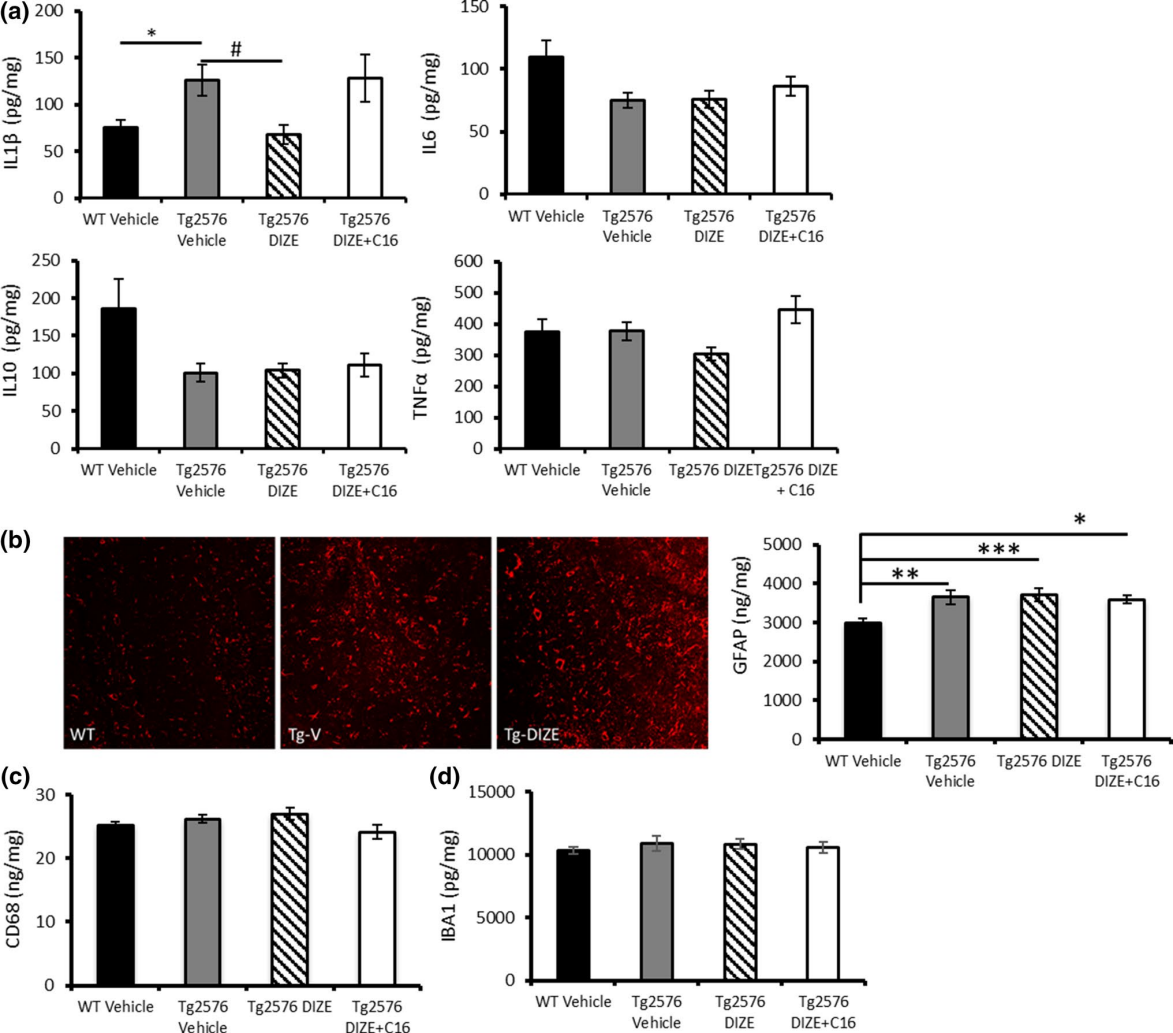
ACE2-mediated restoration of associative recognition memory is associated with reduced IL-1β but not astrocytic or microglial activation. **a** Hippocampal IL-1β level, measured by sandwich ELISA, was significantly increased in Tg2576 vehicle mice (*n* = 22) compared to WT vehicle (*n* = 34) (*p < 0.05) and IL-1β was significantly reduced in Tg2576 DIZE mice (*n* = 21) compared to Tg vehicle mice (^#^*p* < 0.05). IL1-β was not statistically different, however, between Tg vehicle and Tg2576 mice co-administered with DIZE + C16 (*n* = 11) (*p*s > 0.05). No overall change was observed in the level of IL6, IL10 or TNFα between groups (*p*s > 0.05). **b** Representative images of GFAP-positive astrocytes within the hippocampus in WT, Tg-V and Tg-DIZE mice. GFAP level, measured by ELISA, was significantly increased in Tg2576 vehicle mice (*p* < 0.01), Tg2576 DIZE (*p* < 0.001) and Tg2576 DIZE + C16 mice (*p* < 0.05) compared to WT vehicle. **c**, **d** Two independent microglial markers were measured in hippocampal extracts but no overall changes in concentration were observed (*p* > 0.05). Cytokines and markers of activated microglia and astrocytes were quantified by ELISA. Data were analysed using one-way ANOVA with post hoc Tukey analysis. Error bars represent SEM

Hippocampal IL-1β level was significantly altered between groups (*F*(3, 87) = 5.292, *p* = 0.002) and was significantly increased in Tg-V mice compared to WT control (*p* = 0.018) and significantly reduced in Tg-DIZE mice compared to Tg-V mice (*p* = 0.015) (Fig. 5a) (data was obtained from animals in Experiments 1 and 2). Hippocampal IL-1β level in Tg-DIZE + C16 mice was comparable to Tg-V mice (*p* = 1.0) indicating that the reduction in hippocampal IL-1β in Tg-DIZE mice was specifically related to enhanced ACE2 activity. No significant changes were observed in total levels of hippocampal IL6 (*F*(3, 87) = 2.470, *p* = 0.067), IL10 (*F(*3, 87) = 2.053, *p* = 0.113), or TNFα (*F*(3, 87) = 1.555, *p* = 0.206) in Tg2576 mice (Fig. 5a).

Hippocampal reactive astrocytes were immuno-fluorescently labelled with GFAP in the hippocampus and inspection of the sections suggested that they were more abundantly expressed in Tg-V and Tg-DIZE mice compared to WT-V mice (Fig. 5b). Our observations were confirmed by ELISA measurements that showed alterations in GFAP level across groups (*F*(3, 54) = 2.055, *p* = 0.012) with post hoc analysis revealing that GFAP was significantly increased in all Tg groups compared to WT-V: Tg-V (*p* = 0.004); Tg-DIZE (*p* = 0.001) and Tg-DIZE + C16 (*p* = 0.015)) (Fig. 5b). We measured the level of two independent markers of microglia, IBA1 and CD68, in hippocampal homogenates and found no significant difference between Tg2576 and WT mice, or in the treated Tg mice group (ps > 0.05; Fig. 5c, d).

We also measured two independent markers of tissue oxygenation, VEGF and the ratio of MAG:PLP, based on biochemical assays recently developed in postmortem human brain tissue [55, 56]. The level of VEGF (F(3, 54) = 0.24, *p* = 0.87), a marker of ischaemia, and the ratio of MAG:PLP (F(3, 54) = 2.36, *p* = 0.083), a marker of tissue oxygenation that is lowered as a result of reduced perfusion, were both unaltered in the hippocampus across WT and treated Tg mice (Supplementary Fig. 7, online resource).

### DIZE prevented the onset of cognitive impairment and amyloid pathology when delivered to asymptomatic (young) Tg2576 mice

**Fig. 6:**
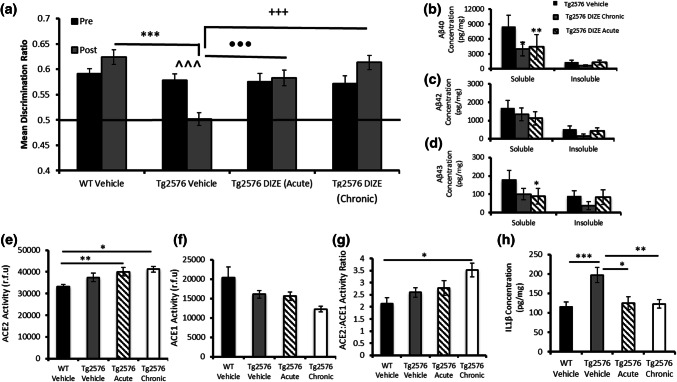
Acute and chronic administration of DIZE prevents the onset of cognitive impairment in young pre-symptomatic Tg2576 mice. **a** Associative recognition memory was assessed in younger WT (*n* = 20) and Tg2576 mice (9–12 months of age) that received either acute (10 days; *n* = 17) or chronic (10 weeks *n* = 11) DIZE administration. Tg2576 vehicle mice (*n* = 16) showed an age-dependent impairment in OiP performance (^^^^^*p* < 0.001), which was not observed in either the acute or chronic DIZE-administered Tg2576 mice (*p*s > 0.05). Tg2576 vehicle mice were also impaired compared to WT vehicle (***p < 0.001), Tg2576 acute (^•••^*p* < 0.001) and Tg2576 chronic DIZE administered mice (^+++^*p* < 0.001). **b**–**d** Aβ levels were quantified by ELISA. Soluble Aβ40 level was reduced in both chronic (*p < 0.05) and acute DIZE-administered Tg2576 mice (**p < 0.01) compared to Tg2576 vehicle mice. Soluble Aβ43 was reduced in Tg2576 mice that received acute DIZE administration (*p < 0.05) compared to Tg2576 vehicle mice. **e**–**g** ACE2 and ACE1 activities were determined by enzyme activity assays. ACE2 activity was significantly increased in the hippocampus of Tg2576 mice administered DIZE either acutely (**p < 0.01) or chronically (*p* < 0.05) compared to WT vehicle mice. Despite a numerical reduction in ACE1 activity in DIZE administered mice, no significant changes were reported, *p* > 0.05; however, the ratio of ACE2:ACE1 was significantly increased in Tg-chronic mice compared to WT vehicle (*p < 0.05). **h** IL-1β level was increased in Tg2576 vehicle mice compared to WT vehicle controls (***p < 0.001) but was significantly reduced after acute (*p < 0.05) and chronic (**p < 0.01) DIZE administration in Tg2576 mice compared to Tg vehicle mice. Behavioural data were analysed using mixed measures ANOVA. Significant interactions were further analysed by tests for simple main effects with Bonferroni corrections for multiple comparisons. ACE2 and IL-1β analyses were performed using one-way ANOVA with post hoc Tukey analysis. Amyloid analysis was performed using non-parametric Kruskal–Wallis one-way ANOVA with multiple comparisons. Error bars represent the SEM

In Experiment 4, contact times (Supplementary Table 4, online resource) and DR scores (Fig. 6a) revealed that 9–10-month-old Tg-V and Tg-DIZE mice were able to discriminate objects in novel locations (i.e. were unimpaired) prior to treatment (ps > 0.9). At 12–13 months of age, when re-tested, Tg-V mice were impaired in the OiP task compared to baseline scores (*p* = 0.001) and compared to WT vehicle mice (*p* = 0.0001). In contrast, Tg2576 mice administered DIZE over a 10-week period starting at 9–10 months of age (asymptomatic) (Tg-DIZE chronic) (*p* = 0.0001) and Tg2576 mice administered DIZE for 10 days at 12–13 months of age (impaired) (Tg-DIZE acute) (*p* = 0.001) both performed significantly better than Tg-V mice and were indistinguishable from WT-V controls mice (ps > 0.1).

In 12–13-month-old Tg-V mice, soluble Aβ levels were considerably higher than insoluble Aβ. Soluble Aβ40 (H(2) = 8.07, *p* = 0.018) and Aβ43 (H(2) = 7.97, *p* = 0.019) level significantly differed in chronic and acute DIZE-treated mice compared to Tg-V (Fig. 6b–d). Post hoc Dunn’s analysis confirmed that soluble hippocampal Aβ40 was significantly reduced in both chronic (51.81%) (*p* = 0.035) and acute (48.93%) (*p* = 0.007) DIZE-treated mice compared to Tg-V. Soluble hippocampal Aβ43 was significantly reduced in acutely treated Tg2576 mice compared to vehicle controls (52.07%) (*p* = 0.014) and numerically reduced by 41.88% in chronic-treated mice (*p* = 0.44). Soluble Aβ42 was numerically reduced in acute-treated (43.25%) (*p* = 0.41) and chronic-treated (28.25%) (*p* = 0.89) mice compared to Tg-V but due to variability between individual mice, the difference was not statistically significant. All percentage changes in acute and chronic DIZE mice are shown in detail in Supplementary Table 5, online resource.

Hippocampal ACE2 activity was significantly higher in Tg-DIZE mice after acute (*p* = 0.010) and chronic (*p* = 0.013) administration compared to WT-V (Fig. 6e). As previously found in older mice, ACE1 was incrementally reduced in relation to duration of DIZE treatment and the ratio of ACE2:ACE1 was significantly higher in Tg-DIZE mice compared to WT-V (p < 0.05) (Fig. 6f–g).

Hippocampal IL-1β level was significantly increased in Tg-V mice compared to WT-V (*p* = 0.001) but was significantly reduced in Tg2567 mice following both chronic (*p* = 0.007) and acute (*p* = 0.016) exposure to DIZE (Fig. 6h).

## Discussion

In the present study, we have shown for the first time that DIZE-mediated enhancement of brain ACE2 activity prevented cognitive decline when administered chronically to young (9–10 months) asymptomatic Tg25676 mice and restored cognitive performance in aged impaired (13–15 months old) Tg2576 mice. Restoration of cognitive performance in older mice was associated specifically with enhancement of ACE2 activity since DIZE-mediated protection was abolished when co-administered with C16, an ACE2 inhibitor. DIZE-mediated ACE2 enhancement was specifically associated with changes in soluble Aβ, rather than plaque-associated insoluble Aβ, within the hippocampus. DIZE, via ACE2, also reduced hippocampal levels of the pro-inflammatory mediator IL-1β. We found that DIZE restored hippocampal MAS receptor expression in Tg2576 mice associated with the evidence of ‘activated’ glutamatergic signalling processes. DIZE also reinstated the balance of hippocampal RAS activity, i.e. elevated ACE2 and reduced ACE1 activity. Together, these novel data identify a modifiable pathway that has not been previously investigated in a transgenic mouse model of Aβ pathology and provide mechanistic insights into a potential novel target for therapeutic application in AD.

We have recently shown that the RAS is imbalanced in early-stage AD; overactivity of classical RAS and reduced activity within regulatory RAS are associated with disease pathology in human AD brains [4, 5, 35]. Regulatory RAS (rRAS) pathways are responsible for Ang-II metabolism and counter-balance the detrimental effects of cRAS [60–62]. The Ang-II metabolite, Ang-(1–7), which is the central signalling peptide in rRAS, is the major angiotensin species in the CNS [22, 23] indicating that rRAS pathways likely dominate in the CNS under normal circumstances. Our recent findings in postmortem AD brain tissue [35, 36] indicate that ACE2 activity was reduced by almost 50% in the mid-frontal cortex in AD [35]. The reduction in ACE2 was strongly related to the levels of parenchymal Aβ and tau in contrast to a weaker relationship between ACE1 and Aβ, where no association with tau was observed [4]. Moreover, in plasma samples from AD patients, Ang-(1–7) level was similarly found to be reduced and related to cognitive performance [63]. Lower levels of Ang-(1–7) were inversely correlated with accumulation of hyperphosphorylated tau in a naturally occurring accelerated ageing mouse line (SAMP8) and in P301S transgenic overexpressing tau mice [64].

The present study importantly demonstrated that ACE2 enhancement, as a result of peripheral DIZE administration, was specifically responsible for improved cognitive performance and reduced Aβ pathology in transgenic Tg2576 mice. DIZE has centrally acting effects indicating that it either crosses the blood–brain barrier (BBB) or at least influences CNS levels of ACE2. DIZE restored cognitive performance in older symptomatic Tg2576 mice and protected against cognitive decline in younger pre-symptomatic mice if administered for 10 weeks in 9–10-month-old asymptomatic mice Tg2576 mice or acutely for 10 days in cognitively impaired 12–13-month-old mice. Our findings are consistent with extensive studies showing cognitive and pathological benefits from enhancing either ACE2 activity or boosting the levels of Ang-(1–7) in mouse models of stroke (reviewed in [65]), chronic cerebral hypoperfusion [66], diabetes [67] and in recent mouse models of AD where compounds were infused intracerebroventricularly [68, 69]. Interestingly, a direct comparison in an experimental rat model of ischemia/reperfusion injury recently indicated that rRAS activation, by XNT-mediated ACE2 enhancement, was more effective at reducing resultant pathology compared to the cRAS blocker telmisartan, a prototypical ARB [30]. DIZE has previously been shown to protect, following intraperitoneal injection for 8 weeks, against cognitive decline and disease-related pathology in a non-transgenic D-galactose-ovariectomized rat model of AD [39]. This is the first report of similar protection in an established transgenic model of AD.

Co-administration of DIZE with C16 (an ACE2 inhibitor) [46, 70] not only allowed us to confirm the cerebroprotective effects of DIZE were mediated by ACE2 but allowed us to identify ACE2-mediated changes that underpinned cognitive protection. For instance, cognitive protection in Tg2576 mice was associated with changes in soluble Aβ species rather than insoluble Aβ load that presumably reflect parenchymal plaque load. It will be important to identify age-related changes in the soluble and insoluble pools and identify the specific Aβ species and assemblies that are associated with ACE2 enhancement and cognitive improvement in future studies. Disease pathology and cognitive function is impaired in ACE2 knockout mice providing further support for a role of ACE2 in AD [71]. DIZE reduced hippocampal ACE1 (cRAS) activity in Tg2576 mice, consistent with our previous findings of an inverse relationship between ACE1 and ACE2 in AD human brain tissue [35] and highlights that rRAS and cRAS are inter-dependent and co-regulated.

The downstream activation of MasR is the likely mechanism preserving cognitive function and reducing amyloid pathology in relation to DIZE administration. Ang-(1–7) has been shown to mediate LTP and synaptic plasticity and improve cognition in rodents following activation of MasR, whereas the protective effects were abolished in MasR KO mice [32, 33]. A meta-analysis recently confirmed the protective role of Ang-(1–7) in cognition [13]. Consistent with this, we observed robust MasR expression within the hippocampus and subiculum in WT mice, as previously shown [31], but found that the expression of MasR was reduced in Tg2576 mice (revealed by IHC and ELISA measurement). Importantly, DIZE restored the expression of MasR in Tg2576 mice within hippocampal synaptosomal extracts. In contrast, the expression of a related alternative rRAS receptor, IRAP, which is downstream of Ang-II metabolism and also enhances cognitive function and LTP [34, 72] remained unchanged. We also found evidence of increased hippocampal expression of a subset of glutamate receptors and signalling pathways, specifically NR2B receptor and ERK, which influence performance on the OiP task performance in normal mice [51, 73]. Activation of ERK, which is downstream of NMDA receptor activity, plays a role in regulating memory processes [74]. Deficiencies in synaptic NMDA NR2B subunit phosphorylation and downstream ERK signalling have previously been associated with behavioural deficits in Tg2576, APP23 and 3xTg mice, due in part to the effects of excessive Aβ [75–77]. We have previously shown that changes in the ratio of total:phosphorylated ERK, specifically within synaptosomes, is associated with OiP performance in PDAPP mice [51]. These data indicate that localised changes in synaptosome ERK signalling observed in the DIZE-administered mice are likely to be indicative of increased signalling through the NR2B receptor. Interestingly, we observed similar changes in ERK phosphorylation in WT mice suggesting that DIZE activates hippocampal glutamate signalling in non-diseased mice, i.e. during normal ageing. Future studies should address whether DIZE promotes synaptic and cognitive processes in aged WT mice.

We explored the possible mechanisms that might be associated with DIZE-mediated reduction in disease-related pathology and improved cognition in Tg2576 mice. Overactivation of cRAS is associated with oxidative stress, inflammation, and vascular dysfunction that are likely to contribute to disease pathology and cognitive impairment in AD (reviewed in [2]). Of the four cytokines studied (IL-1β, IL-6, IL-10 and TNF-α), we found that hippocampal IL-1β, elevated in Tg2576 mice, was lower and comparable to WT levels following DIZE treatment. As for soluble Aβ, reduced IL-1β was specific to ACE2 enhancement (i.e. the effect was not observed in DIZE + C-16 mice) and was specifically associated with restoration and protection against cognitive decline. IL-1β is a pro-inflammatory cytokine that is elevated in AD (reviewed in [78]) and has previously been shown to impair LTP and learning and memory performance in rodents [79, 80]. Signalling through the MasR (by Ang-(1–7)) exerts anti-inflammatory effects, including reducing IL-1β mRNA levels in cultured microglia [81], which could provide an explanation for the observed protective effects of DIZE. RAS activity, particularly cRAS activation, has also been proposed as an important ‘switch’ in promoting a pro-inflammatory M1 state, whilst rRAS activity favours an immune-suppressive ‘phagocytic’ M2 phenotype [82], which could provide a mechanistic explanation for the removal of Aβ [83, 84].

Whilst an attractive hypothesis, our analysis has failed to detect any significant changes in two independent markers of microglial activation (CD68 and IBA1)—a major source of IL-1β. However, a more comprehensive analysis of different sub-types of microglial activation markers (particularly markers of M1/M2 phenotype) is required. Comprehensive analysis of these inflammatory markers in WT mice would also provide additional insight into the role of DIZE in mediating cerebroprotection in healthy aged mice and should be a focus of future studies. Astrocytes were constitutively activated in Tg-2576 mice and remained activated in the presence of both DIZE and DIZE + C16 indicating that astrocyte activation is unlikely to contribute to the observed cognitive protection in Tg2576 mice at least at this age. The protective effect of DIZE was also not associated, again somewhat surprisingly, given the role of RAS in vaso-modulation and blood pressure, with changes in the expression of markers of brain tissue perfusion, VEGF and MAG:PLP (as reviewed in [85]) but this is consistent with our findings of unchanged MABP throughout the study. Given the recent evidence that RAS-targeting ARBs and ACE-Is, associated with reduced incidence and delayed onset of cognitive decline in AD patients, are associated with reduced tau pathology and lower CSF tau level [14, 86, 87], as well as the ability of the ARB losartan to reverse Ang-II-mediated tau phosphorylation in aged rodents [8], further studies should address whether DIZE also modifies disease-related tau modifications in relation to cognitive decline.

Despite the highly probable involvement of the rRAS pathway in mediating the protective effects of DIZE, we should acknowledge that we did not find an increase in Ang-(1–7) levels in DIZE-treated mice. This may be due to inherent technical difficulties in measuring the level of Ang-(1–7) within brain tissue due to low expression and the short half-life of the peptide; however, it could also indicate the involvement of other rRAS peptides, such as angiotensin-(1-9), that could also potentially activate MasR but which were not measured in this study. Additionally, DIZE-mediated ACE2 activity could act independently of or in combination with rRAS. For instance, ACE2 converts Aβ43, an early-depositing species of Aβ that has been postulated to be an important seed for parenchymal plaque growth and is elevated in AD [88], to a less toxic Aβ species via sequential cleavage of Aβ43 to Aβ42 followed by ACE1-mediated cleavage to the less toxic Aβ40 [89]. Thus, DIZE-mediated protection might help promote clearance of this highly amyloidogenic early-depositing form of Aβ. Further studies are, therefore, required to determine the precise pathway/s that underly the protective effects of DIZE before we can begin to translate these promising preclinical findings into the clinic. Given the established influence of gender on RAS activation [90–92], particularly in post-menopausal females, it will also be important to determine if the effects of DIZE are replicated in female mice to inform which patient groups are likely to benefit from rRAS intervention.

Collectively these data indicate the potent cognitive and pathological benefits following DIZE-mediated ACE2 enhancement in a Tg2576 mouse model of AD. Our findings, together with recent data from postmortem brain tissue, reaffirm the importance of the angiotensin hypothesis in AD and indicate that activation of the regulatory ACE2/Ang-(1–7)/MasR rRAS pathway, which works to downregulate the cRAS pathway whilst directly boosting memory and learning, provides an exciting, alternative and novel therapeutic target for potential treatment in AD.

## Electronic supplementary material

Below is the link to the electronic supplementary material.

Supplementary material 1 (DOCX 11315 kb)
