# Supplementary material for: ACE2 activation protects against cognitive decline and reduces amyloid pathology in the Tg2576 mouse model of Alzheimer’s disease
Source: Acta Neuropathol. 2020 Jan 25;139(3):485–502. doi: 10.1007/s00401-019-02098-6 (PMC7035243; doi:10.1007/s00401-019-02098-6)
Supplement: Supplementary file 1 — Supplementary material 1 (DOCX 11315 kb) [file 401_2019_2098_MOESM1_ESM.docx]

**Supplementary Figures**

**Supplementary Figure 1. Schematic illustration of the object-in-place task used to test associative recognition memory in this study**. Animals are presented with 4 unique objects in the sample phase. Tg2576 mice were given 10-minutes to freely explore these objects followed by a 2-minute retention interval prior to the test phase. In the test phase the spatial arrangement of 2 of the objects was switched and the time animals spent exploring the objects in familiar and novel arrangements was recorded. WT mice were “yolked” to Tg2576 mice in the sample phase as previously described (Good and Hale, 2007; Hale and Good, 2005). Briefly, WT mice were randomly paired with Tg2576 mice. During the sample phase, Tg2576 mice were given the full 10-minutes to explore objects. The paired WT mouse was then allowed to equal this total contact time of its partnered Tg2576 mouse or given the full 10-minutes sample trial, whichever was reached first. However, all mice were given 10-minutes at test phase. Discrimination ratios were calculated as time spent exploring objects in novel arrangements/total time spent exploring all 4 objects. For all studies, at both baseline and final assessment point, mice were tested twice on the OiP task and the scores were averaged to minimize individual variability. All object sets were counterbalanced across groups at individual time-points to negate the potential confounding effects of repeated measures.

**
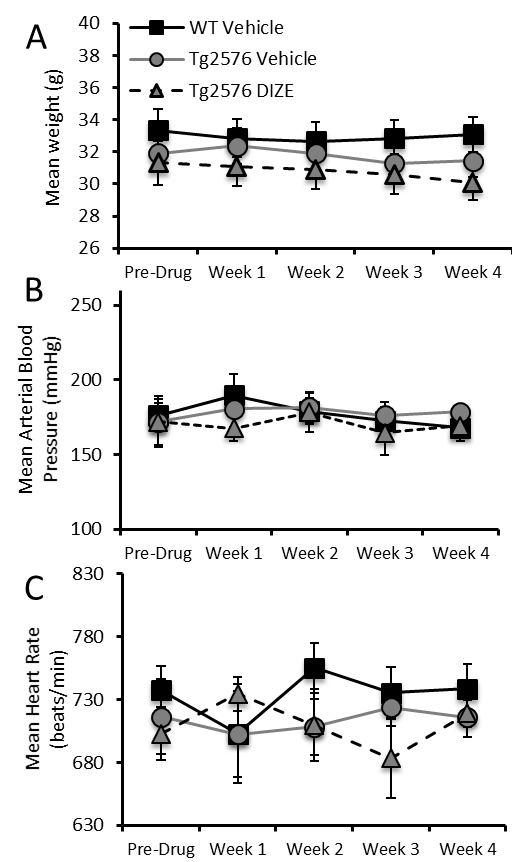
**

c

b

a

**Supplementary Figure 2. Restoration of cognitive performance in DIZE-treated Tg2576 mice is not associated with changes in major physiological parameters.** No significant change in weight **(a),** mean arterial blood pressure (MABP) **(b)** or heart rate **(c)** was observed in DIZE-treated Tg2576 mice over the 30-day period compared to Tg2576-Vehicle and WT-Vehicle mice (p’s>0.05) (n=6/group). Data were analyzed using mixed-measures ANOVA. Between and within-subject comparisons were performed with Bonferroni corrections for multiple comparisons. Error bars represent the standard error of the mean (SEM).


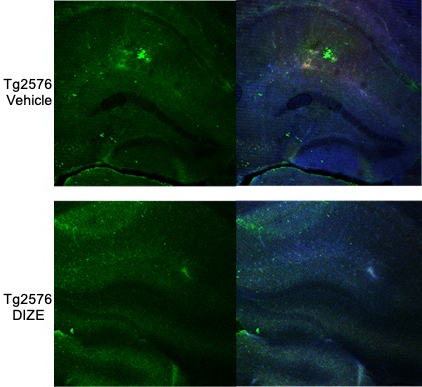


**Supplementary Figure 3.** **DIZE administration reduces Aβ plaque pathology in the hippocampus of Tg2576 mice.** Representative images of Aβ plaque pathology in the hippocampus of 14-15 month old Tg Vehicle (upper panel) and Tg mice administered daily with DIZE by IP (15mg/kg/day) (lower panel). Sections were labelled with anti-human Aβ (4G8) (green) and counterstained with DAPI (blue). Images were taken under x20 magnification.


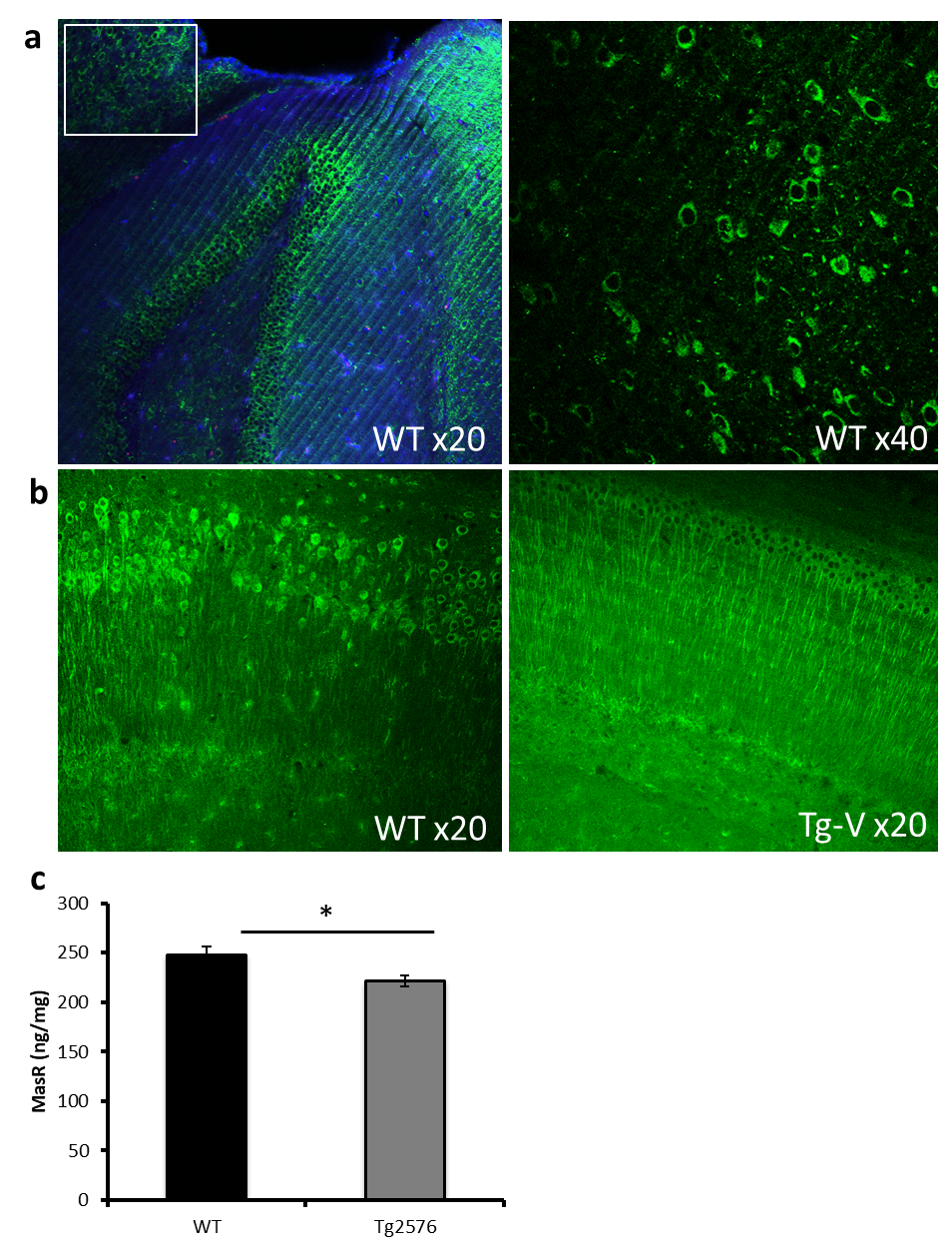


**Supplementary Figure 4.** **Hippocampal MasR expression is reduced in Tg-2576 mice. (a)** Representative images of hippocampal expression of MasR within the dendate gyrus and subiculum in WT (C7Bl/6) mice (x20 and x40 shows enlarged view of white box) **(b)** Representative images comparing the expression of MasR in WT-V and Tg-2576 mice within the CA4 region of the hippocampus. **(c)** MasR levels in hippocampal extracts, measured by sandwich ELISA, were significantly lower in Tg2576 mice (n = 9) compared to WT mice (n = 11) (p<0.05). Data were analyzed using independent samples t-test. Error bars represent the SEM.

**Supplementary Figure 5. Changes in hippocampal synaptosomal proteins.** Full blots are shown for **(a)** Total NR2B and phosphorylated tyrosine residue 1472. **(b)** Total GluA1, GluA1 phosphorylated at serine 845 and Mas Receptor. **(c)** IRAP receptor and PSD95. **(d)** Total ERK and phosphorylated ERK. Analysis of specific band densities are shown in Figure 4 within the main manuscript.

**Supplementary Figure 6. No overall changes in Tau phosphorylation were observed in Tg2576 mice following DIZE administration.** **(a)** Representative western blot of hippocampal synaptosomes showing total tau levels and tau phosphorylated at serine 396/404 (PHF-1). **(b)** No overall changes were shown in either total tau or tau phosphorylated at serine 396/404 (PHF-1 epitope) when protein levels were quantified.

b

a

**Supplementary Figure 7.** Markers of tissue oxygenation and ischemia are unchanged in Tg2576 mice following DIZE-administration. **(a)** VEGF level, a marker of brain ischemia, measured by sandwich ELISA, was unaltered between WT, Tg-V and Tg-DIZE mice (*F*(3, 54)=0.24, p=0.087 **(b)** A biochemical marker of tissue perfusion, the ratio of MAG:PLP that indicates tissue oxygenation, was unchanged in WT and Tg-V/Tg-DIZE mice *F*(3, 54)=2.36, p=0.083. Data were analyzed using mixed-measures ANOVA. Between and within-subject comparisons were performed with Bonferroni corrections for multiple comparisons. Error bars represent the standard error of the mean (SEM).

a
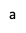


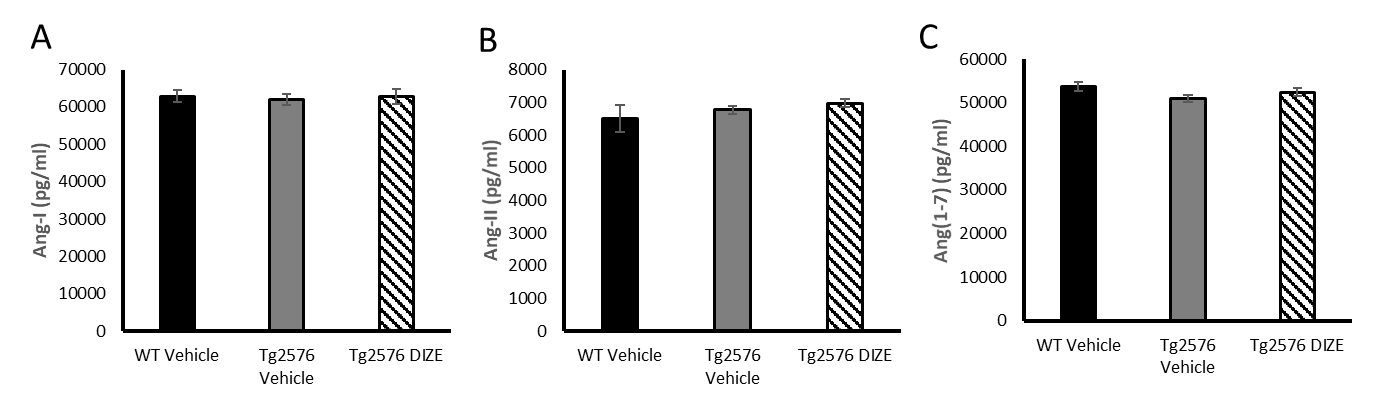


c
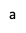


b
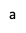


**Supplementary Figure 8. Angiotensin peptide levels in DIZE-treated Tg2576 mice are unaltered. (a-c)** Hippocampal Ang-I, Ang-II and Ang-(1-7) peptide concentrations, measured by direct ELISA, were unchanged in WT (n=12) and Tg2576-Vehicle (n=11) and Tg2576-DIZE (n=9) treated mice, p’s>0.05. Data were analysed using one-way ANOVA. Error bars represent the SEM.

**Supplementary Tables:**

**Supplementary Table 1**

|  | | Mean Contact Times (s) | | |
| --- | --- | --- | --- | --- |
|  |  | Sample Phase | Test Phase | |
| Group | Pre/Post Treatment |  | Familiar | Novel |
| WT Vehicle | Pre | 49.86 (19.16) | 29.01 (8.94) | 44.86 (14.84) |
|  | Post | 53.62 (13.93) | 32.80 (15.69) | 47.77 (16.47) |
| Tg2576 Vehicle | Pre | 53.87 (20.07) | 31.63 (12.10) | 31.40 (12.25) |
|  | Post | 58.65 (20.10) | 31.04 (15.19) | 30.89 (14.34) |
| Tg2576 DIZE | Pre | 51.55 (25.11) | 27.61 (12.87) | 30.93 (13.83) |
|  | Post | 53.02 (21.08) | 18.24 (9.42) | 27.29 (13.05) |

**Supplementary Table 1:** Mean contact times with objects in the sample phase and test phase during the object-in-place task for animals from Cohort 1 at baseline (Pre) and following 30-days DIZE administration (Post) are shown. The standard deviation of each mean is shown in brackets.

*Sample Phase:*

Supplementary table 1 shows the mean contact times with objects in sample and test phases. Previous studies with Tg2576 mice showed WT’s explored objects more in sample phases compared to Tg2576 mice (Hale and Good, 2005). For this reason, WT mice were yoked to a pair of Tg2576 mice in both pre- and post-treatment time points (as explained in the methods and in (Hale and Good, 2005)). Analysis of the sample phase contact times confirmed neither group of mice explored objects significantly more, *F*(2, 29)=0.21, p=0.82 as a result of the yolking procedure i.e. WT mice did not perform any better in test trials due to increased contact times with objects in the sample phase.

*Test Phase:*

Despite Tg2576 mice displaying lower total contact times with objects in both pre- and post-treatment stages, there was no main effect of group, *F*(2, 29)=3.08, p=0.061. A significant object location x group *F*(2, 29)=40.13, p=0.0001 interaction revealed that (when data were collapsed across time) WT mice explored objects in novel locations more than Tg2576 vehicle, p=0.017 and Tg2576 DIZE mice, p=0.012. No difference in exploration of objects in novel locations between Tg2576 groups was reported, p=1.0. No difference in contact time with familiar objects was observed (minimal p=0.332 (Tg2576 vehicle vs Tg DIZE). WT vehicle and Tg2576 DIZE mice explored objects in novel locations more than familiar (p’s=0.0001), however Tg2576 vehicle mice showed no preference, p=0.89. This ability for DIZE administered Tg2576 mice to explore objects in novel locations in preference to familiar is likely an effect from post-treatment scores. However, no significant group x object location x time was reported *F*(2, 29)=1.20, p=0.32.

**Supplementary Table 2:**

|  | | Mean Contact Times (s) | | |
| --- | --- | --- | --- | --- |
|  |  | Sample Phase | Test Phase | |
| Group | Pre/Post Treatment |  | Familiar | Novel |
| WT Vehicle | Pre | 38.93 (17.46) | 25.40 (13.65) | 38.79 (19.41) |
|  | Post | 30.13 (11.02) | 22.33 (8.45) | 39.14 (18.58) |
| Tg2576 Vehicle | Pre | 41.10 (24.95) | 17.53 (9.29) | 19.85 (10.26) |
|  | Post | 37.14 (17.20) | 17.83 (9.08) | 18.32 (7.76) |
| Tg2576 DIZE | Pre | 34.02 (19.21) | 15.69 (12.63) | 16.14 (11.39) |
|  | Post | 30.16 (25.69) | 15.34 (15.21) | 22.53 (16.42) |
| Tg2576 DIZE + C16 | Pre | 38.46 (24.07) | 14.44 (4.79) | 16.50 (7.31) |
|  | Post | 23.80 (7.43) | 14.26 (10.41) | 14.50 (9.65) |

**Supplementary Table 2:** Mean contact time scores at pre- and post-treatment stages for WT vehicle mice and Tg2576 mice administered either vehicle, DIZE or DIZE+C16. Standard deviation is shown in brackets.

*Sample Phase*

As initially reported, sample phase analysis showed no overall difference between groups in total contact time with objects, *F*(3, 52)=0.68, p=0.568. Despite a main effect of treatment, *F*(1, 52)=15.443, p=0.0005 indicating an overall reduction in contact time at the pre-treatment phase, no group x treatment interaction was reported, *F*(3, 52)=1.139, p=0.342, indicating this change in contact time was similar across all groups.

*Test Phase*

As already reported above, WT mice appeared to explore objects more than Tg2576 mice in the test phase *F*(3, 52)=7.194, p=0.0005. Post-hoc Tukey analysis revealed WT’s explored objects more than Tg2576 vehicle (p=0.035), Tg DIZE (p=0.004) and Tg2576 DIZE + C16 mice (p=0.002; when data were collapsed across treatment intervention). Analysis of the contact times recorded in the OiP test phase revealed a significant group x treatment x object location interaction, *F*(3, 52)=4.084, p=0.011. Tests for simple main effects revealed that WT mice explored objects in novel locations more than familiar locations at both the pre- (p=0.001) and post-treatment (p=0.001) time points. In contrast, both Tg2576 Vehicle and Tg2576 DIZE + C16 showed no preference of objects in novel locations at either pre- (p=0.090 and 0.134) or post-treatment (p=0.757 and 0.779). However, Tg2576 mice administered DIZE only showed an improved overall performance. Pre-treatment analysis showed no preference to explore objects in novel locations (p=0.557), whilst following DIZE administration, Tg2576 mice showed a preference to explore objects in novel locations of familiar (p=0.001). Collectively these data indicate that whilst DIZE administration and enhanced ACE2 activity does not have an effect on changing the overall contact time with objects in Tg2576 mice, it does improve the performance of Tg2576 mice to explore objects in novel locations in preference to familiar.

**Supplementary Table 3:**

|  | Mean Contact Times (s) | | |
| --- | --- | --- | --- |
|  | Sample Phase | Test Phase | |
| Group |  | Familiar | Novel |
| WT Vehicle | 28.20 (12.32) | 20.85 (10.88) | 34.95 (19.00) |
| WT DIZE | 33.51 (8.12) | 20.05 (12.03) | 32.14 (14.84) |
| Tg2576 Vehicle | 33.96 (20.17) | 21.01 (15.38) | 22.67 (17.80) |
| Tg2576 DIZE | 36.85 (17.26) | 13.71 (7.66) | 22.45 (15.05) |

**Supplementary Table 3:** Mean contact times with objects in the sample phase and test phase during the object-in-place task in cohort 3. The standard deviation of each mean is shown in brackets.

Cohort 3 Contact times Analysis:

Analysis of contact time data (Supplementary Table 3) showed that no difference in sample phase contact times, *F*(3, 27)=0.39, p=0.76 were reported. In the test phase, a significant main effect of object location was reported in this analysis, *F*(1, 24)=31.48, p=0.0001, whereby mice had a higher contact time with objects in novel locations compared to familiar. This appeared different between groups, confirmed by a significant group x location interaction, *F*(3, 24)=3.108, p=0.045. Tests for simple main effects confirmed that objects in novel locations were explored more than familiar by WT-vehicle (p=0.0001), WT DIZE (p=0.001) and Tg2576 DIZE (p=0.004), but not by Tg2576-vehicle mice (p=0.655). These data further contribute to findings in cohort 1 and 2 and indicated that DIZE reverses the OiP impairment in Tg2576 mice and does not further alter performance in WT mice.

Test Phase DR Scores:

Significant difference in DR scores, *F*(3, 27)=4.77, p=0.010. Significant reduction in OiP performance in Tg2576 vehicle mice compared to WT vehicle (p=0.044), WT DIZE (p=0.010). Although reduced compared to Tg2576 DIZE, this was not significant (p=0.052). No further group differences were reported. One-sample t-tests were used to confirm mice performed significantly above chance. All mice except Tg2576 vehicle were significantly above chance (minimal effect Tg2576 DIZE mice t(6)=3.31, p=0.016). Tg2576 vehicle mice were not above chance performance t(6)=0.50, p=0.64 and were, therefore, impaired on this task compared to all other groups.

**Supplementary Table 4:**

|  | | Mean Contact Times (s) | | |
| --- | --- | --- | --- | --- |
|  |  | Sample Phase | Test Phase | |
| Group | Pre/Post Treatment |  | Familiar | Novel |
| WT Vehicle | Pre | 50.33 (12.72) | 32.39 (7.46) | 47.98 (14.39) |
|  | Post | 44.82 (15.42) | 29.47 (9.35) | 50.02 (13.55) |
| Tg2576 Vehicle | Pre | 55.93 (18.39) | 26.78 (10.33) | 37.73 (17.94) |
|  | Post | 37.44 (21.12) | 24.48 (20.01) | 26.16 (23.44) |
| Tg2576 Chronic | Pre | 46.93 (22.26) | 25.15 (15.99) | 30.74 (15.58) |
|  | Post | 39.77 (24.00) | 19.08 (12.83) | 29.22 (15.97) |
| Tg2576 DIZE Acute | Pre | 47.23 (29.15) | 24.74 (19.38) | 31.89 (23.08) |
|  | Post | 42.37 (23.29) | 21.30 (13.29) | 27.89 (18.14) |

**Supplementary Table 4:** Mean contact times with objects for WT and Tg2576 mice administered DIZE for Chronic (10-week) and Acute (10-day) treatment periods. Numbers in () represent the standard deviation.

*Sample Phase*

Sample phase analysis of mice administered DIZE over acute and chronic periods showed no overall difference between groups in total contact time with objects, *F*(3, 60)= 0.348, p=0.790. However a main effect of treatment, *F*(1, 60)=21.384, p=0.000 indicated an overall reduction in contact times with objects in the post-treatment phase. Despite this effect appearing a common trend within groups, a group x treatment interaction, *F*(3, 60)=3.269, p=0.027 further indicated this was mostly an effect observed within the Tg2576 vehicle mice when comparing their pre- and post-treatment contact times (p=0.001).

*Test Phase*

Analysis of total contact times with objects in the test phase further saw a significant main effect of group *F*(3, 60)=4.159, p=0.010 as previously reported above. WT mice showed a general trend to explore objects more than Tg2576 groups, however this effect was only significant in Tg2576 administered DIZE over a 10-day period (acute; p=0.014). When data were assessed to determine any changes in contact times with objects n novel and familiar locations there was a significant group x treatment x object location interaction, *F*(3, 52)=9.506, p=0.000. Test for simple main effects showed that at the pre-treatment phase, all mice explored objects in novel locations in preference to familiar (all p’s<0.01). However, following vehicle or DIZE treatment WT mice continued to show a preference to explore novel arrangements more than familiar (p=0.000). This effect was also maintained in Tg Chronic (p=0.000) and Tg Acute mice (p=0.000). However, Tg vehicle mice failed to show a preference to novelty at post-treatment assessment (p=0.583). This effect was further confirmed by a significant increase in the total time spent exploring objects in novel locations by chronic and acute DIZE administered mice compared to the time Tg2576 vehicle mice spent exploring objects in novel arrangements (p=0.043 and 0.004).

When contact times were expressed as DR scores, a main effect of group (collapsed across treatment periods) was reported, *F*(3, 60)=6.718, p=0.001. Post-hoc Tukey analysis showed that Tg2576 vehicle mice were significantly impaired compared to WT vehicle mice (p=0.000) and Tg2576 mice that received DIZE treatment over a 10-week period (chronic; p=0.047). No further significant differences were reported (maximal effect Tg2576 vehicle vs Acute treatment, p=0.077).

Further to this, a significant group x treatment interaction was also reported, *F*(3, 60)=10.83, p=0.000. Tests for simple main effects showed that, pre-treatment, no group differences were reported (all p’s>0.9). However, following treatment, Tg2576 vehicle mice showed a significant decline in performance, p=0.001, and were significantly impaired compared to WT vehicle (p=0.000), Tg2576 Chronic (p=0.000) and Tg2576 Acute treatment groups (p=0.001). Moreover, WT vehicle mice showed no difference in DR scores compared to either Tg2576 chronic (p=1.00) or acute (p=0.178) DIZE administered mice. No further differences were reported.

**Supplementary Table 5:**

|  | | Percentage Amyloid Reduction (Relative to Tg2576 Vehicle) | | | |
| --- | --- | --- | --- | --- | --- |
|  |  | Cohort 1+ 2 | | Cohort 4 | |
| Amyloid Type | | DIZE | DIZE+C16 | DIZE Acute | DIZE Chronic |
| Aβ40 | Soluble | 35.59 (50.04) | 3.09 (86.51) | 48.93 (101.19) | 51.81 (50.01) |
|  | Insoluble | 39.69 (51.73) | 46.93 (53.09) | 10.10 (128.85) | 51.33 (29.00) |
| Aβ42 | Soluble | 38.86 (74.81) | 44.54 (60.95) | 43.25 (72.96) | 28.25 (63.57) |
|  | Insoluble | 55.42 (55.45) | 61.79 (45.26) | 21.34 (140.80) | 69.57 (72.79) |
| Aβ43 | Soluble | 37.11 (51.30) | 0.55 (45.45) | 52.07 (92.32) | 41.88 (57.39) |
|  | Insoluble | 44.88 (44.44) | 64.69 (30.95) | 8.69 (151.63) | 55.90 (75.79) |

**Supplementary Table 5:** Mean percentage reduction of Aβ species in mice administered DIZE across cohorts 1&2 combined and cohort 4. Data represent the percentage change relative to the Tg2576 vehicle control group. In brackets represents the standard deviation.

**References:**

Good, M. a, Hale, G., 2007. The “Swedish” mutation of the amyloid precursor protein (APPswe) dissociates components of object-location memory in aged Tg2576 mice. Behav. Neurosci. 121, 1180–91. https://doi.org/10.1037/0735-7044.121.6.1180

Hale, G., Good, M., 2005. Impaired visuospatial recognition memory but normal object novelty detection and relative familiarity judgments in adult mice expressing the APPswe Alzheimer’s disease mutation. Behav. Neurosci. 119, 884–91. https://doi.org/10.1037/0735-7044.119.4.884
